# Phylogeographic inference of Sumatran ranids bearing gastromyzophorous tadpoles with regard to the Pleistocene drainage systems of Sundaland

**DOI:** 10.1038/s41598-022-14722-9

**Published:** 2022-07-19

**Authors:** Umilaela Arifin, Utpal Smart, Martin Husemann, Stefan T. Hertwig, Eric N. Smith, Djoko T. Iskandar, Alexander Haas

**Affiliations:** 1 Centre for Taxonomy and Morphology, Leibniz Institute for the Analysis of Biodiversity Change, Martin-Luther-King-Platz 3, 20146 Hamburg, Germany; 2 Universität Hamburg, Edmund-Siemers-Allee 1, 20148 Hamburg, Germany; 3 Department of Biology, Texas State University, 601 University Drive, San Marcos, TX 78666 USA; 4 Amphibian & Reptile Diversity Research Center Department of Biology, University of Texas at Arlington, Arlington, TX 76019-0498 USA; 5 Naturhistorisches Museum der Burgergemeinde Bern, Bernastrasse 15, 3005 Bern, Switzerland; 6 Institute of Ecology and Evolution, University of Bern, Baltzerstrasse 6, 3012 Bern, Switzerland; 7 Basic Science Committee, Indonesian Academy of Sciences, Jalan Medan Merdeka Selatan 11, Jakarta, 10110 Indonesia

**Keywords:** Evolution, Genetics, Molecular biology, Zoology

## Abstract

Rivers are known to act as biogeographic barriers in several strictly terrestrial taxa, while possibly serving as conduits of dispersal for freshwater-tolerant or -dependent species. However, the influence of river systems on genetic diversity depends on taxa-specific life history traits as well as other geographic factors. In amphibians, several studies have demonstrated that river systems have only minor influence on their divergence. Here, we assess the role of the paleodrainage systems of the Sunda region (with a focus on the island of Sumatra) in shaping the evolutionary history of two genera of frogs (*Sumaterana* and *Wijayarana*) whose tadpoles are highly dependent on cascading stream habitats. Our phylogenetic results show no clear association between the genetic diversification patterns of both anurans genera and the existence of paleodrainage systems. Time-calibrated phylogenies and biogeographical models suggest that these frogs colonized Sumatra and diversified on the island before the occurrence of the Pleistocene drainage systems. Both genera demonstrate phylogenetic structuring along a north–south geographic axis, the temporal dynamics of which coincide with the geological chronology of proto Sumatran and -Javan volcanic islands. Our results also highlight the chronic underestimation of Sumatran biodiversity and call for more intense sampling efforts on the island.

## Introduction

Sundaland (the combined landmasses that comprise the Malay Peninsula, Borneo, Sumatra, and Java, and the shallow sea in between) is a global biodiversity hotspot with prodigious amount of biodiversity and a large number of local endemics^1–3^. This diversity and high rate of endemism have primarily been accredited to the dynamic abiotic history of the region especially during the Cenozoic^4,5^. The geological events during the Cenozoic caused different configurations of land and sea over time and this, in turn, has impacted the climate, vegetation, and the availability of its faunal habitats^6–8^. Several studies^9–13^ have investigated the historical processes of Sundaland to explain species diversity and its unique distribution patterns in the region today.

Despite recurrent sea-level fluctuations, most of the western parts of Sundaland were subaerial from the Eocene to the Early Miocene, with some evidence for the existence of large freshwater lakes^14,15^. Volcanic arcs also formed at the southern margin of the Sunda region during this period^14,16^. The volcanic activity in Sumatra became more extensive from the Mid Eocene because of regional subsidence^14,16,17^. In the Quaternary period of the Last Glacial Maximum (LGM), the sea level was recorded at its lowest (120 m below present) and the climate was considerably cooler and drier^8^. However, it has been suggested that during much of the Quaternary the climate was presumably neither wetter nor drier than during the LGM^8^. Sea-level oscillations during this time periodically established connections between the different landmasses of the Sunda Shelf^18^ and formed four extensive paleoriver systems in the Sunda and Sahul shelves, i.e., the Malacca Strait River System, the Siam River System, the North Sunda River System, and the East Sunda River System^19,20^. These Pleistocene drainage systems purportedly had impacts on the biodiversity patterns in the region as they could have served as potential dispersal routes between the Greater Sunda Islands^19,20^. For example, the freshwater riverine faunas of rivers presently restricted to Indo-China, the Malay Peninsula, or one of the Greater Sunda Islands were probably connected during the Pleistocene^11,19,21,22^.

Extant species can be mapped onto the river systems and analyzed with biogeographic and/or phylogeographic approaches. The genetic structure of populations may have been strongly influenced by dispersal barriers resulting from fragmentation of the riverscape (defined as a mosaic of freshwater river habitat that is spatially structured and hierarchically organized across multiple scales^23^). Genetic diversity may be partitioned and regionally distributed according to river drainages if the paleoriver systems played a significant role in the speciation and dispersal of the groups of organisms under study. This appears to be the case, at least for some freshwater organisms and other groups with strong aquatic dependencies. For example, de Bruyn et al.^12^ found a strong correlation between phylogenetic structuring in clades of freshwater fishes and their distributions across Sundaland paleodrainages. Similar phylogeographic patterns have been observed in other taxa such as southern Indochinese amphibians^24^ of the Lower Mekong in southern Indochina, and the Mekong mud snake^25^. The evidence from these studies suggests that the Quaternary landscapes of Indochina and the Sunda Shelf shaped the genetic divergence patterns in populations of certain taxa. The influence of rivers (either as corridors or barriers) on the distribution and genetic structure of local fauna has also been tested in various other taxa and regions (e.g., in the Amazon: frogs^26^, frogs and small mammals^27^, mammals^28^, birds^29^; on Madagascar: mammals^30^, frogs^31^; in the southern USA: fish^32^; in eastern Australia: water skinks^33^). These studies showed that the vicariant influence of river systems depends on taxon-specific life-history traits as well as the geographic setting^31^. Moreover, the current hydrography of river systems may not always explain observed species distribution patterns.

Most amphibians undergo a complex biphasic life cycle with larval forms that are restricted to and strongly depend on aquatic habitats, whereas the terrestrial stages are potentially more vagile and prone to dispersal. Still, rivers may serve as barriers in amphibian populations and may confine their dispersal^34–36^. Here we selected two endemic genera of Sumatran ranid frogs (*Sumaterana* and *Wijayarana*) to investigate whether the paleoriver systems of Sumatra played a role in structuring the distribution of their genetic diversity. Given the trans-island distribution of *Wijayarana*, we also included samples representing populations from the western part of Java. Species of *Sumaterana* and *Wijayarana,* the only Sumatran ranids that inhabit torrential streams, possess gastromyzophorous tadpoles^37–39^ that are characterized by the possession of a large adhesive sucker at the abdomen as an adaptation to torrential stream habitats^40^. Because of their strong association with cascading streams during their larval stage, *Sumaterana* and *Wijayarana* species appear as suitable focal taxa to assess the role of paleodrainage systems of the Sunda region, in shaping the phylogenetic structure of regional stream-dependent anurans on the Sunda region. We particularly focused on Sumatra given that four major paleodrainage systems of Sundaland concurrently existed on this island^19^. Our results show that the distribution patterns of *Sumaterana* and *Wijayarana* do not correlate with the Quaternary paleodrainage systems of Sumatra. Instead, the diversification processes of these taxa appear to be more complex, with the focal taxa having colonized Sumatra and diversified therein much earlier than the formation of the Pleistocene River systems. More specifically, the spatiotemporal diversifications of both genera are best explained by the orogenesis of the Bukit Barisan mountain range and initial episodes of geographic isolation of landmasses (between the Miocene to Pliocene) that form the contemporary volcanic islands in the region.

## Result

### Phylogenetic relationships of Sumatran ranids with gastromyzophorous tadpoles

**Figure 1:**
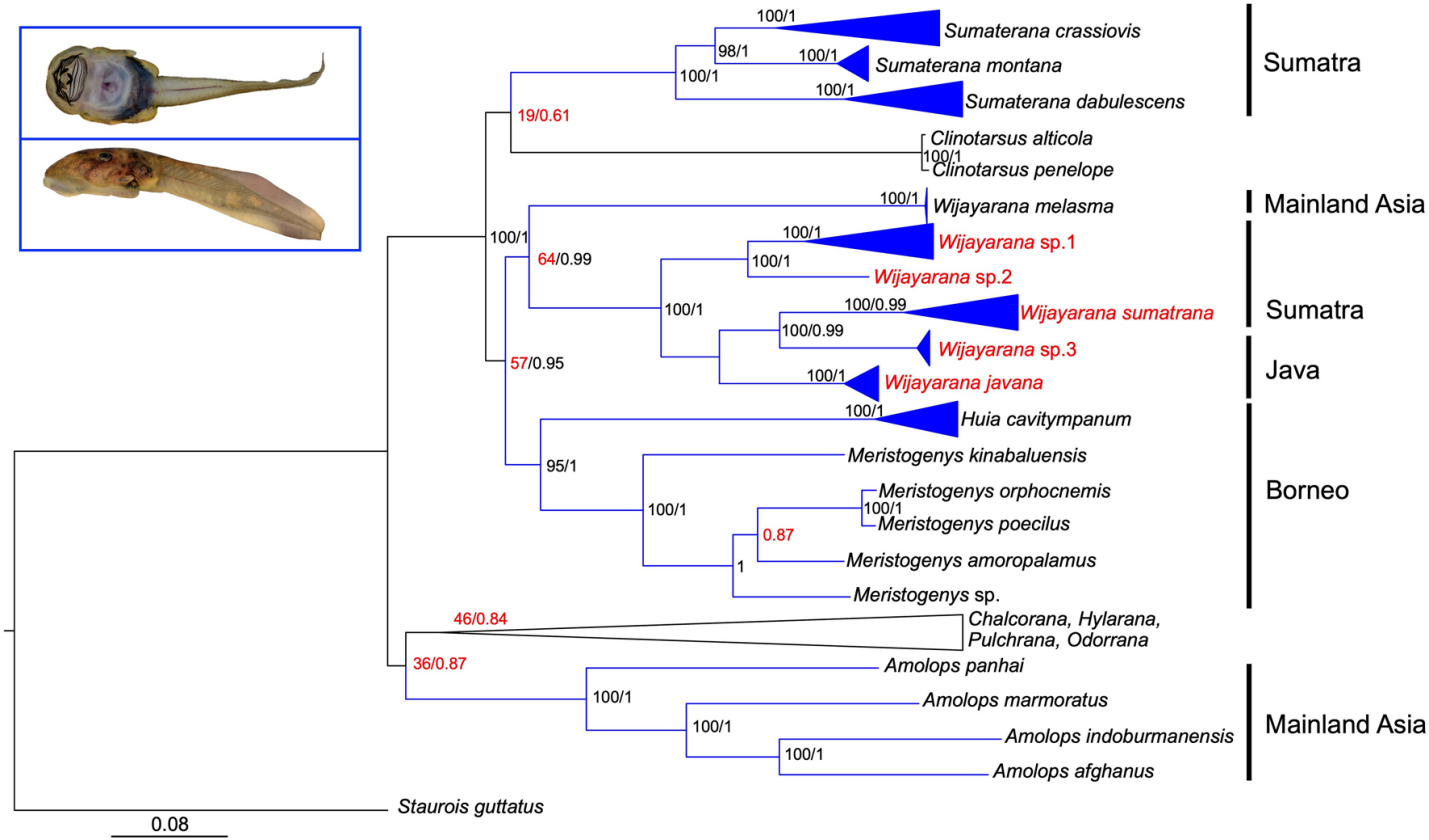
BI tree showing phylogenetic relationships of the Sumatran ranids with gastromyzophorous tadpoles (blue branches) within the family Ranidae. Values denote bootstrap and posterior probabilities (BS/PP). For node between *Meristogenys amoropalamus* and *M. orphocnemis* + *M. poecilus* and the node between this clade and *Meristogenys* sp, only PP value. Red taxa represent the five distinct lineages of *Wijayarana* from Sumatra and Java. Morphology of gastromyzophorous tadpoles (ventral and lateral view; photos by UA) is shown on the upper left.

We generated a new sequence dataset (N = 146; sampling localities shown in Fig. S1), comprising ten concatenated genetic loci (mtDNA and nucDNA) for Sumatran frogs of the genera *Sumaterana* and *Wijayarana* along with sequences of closely related taxa within Ranidae (Supplementary Table S1). We used the final concatenated alignment of a total of 7,582 bp (28.91% proportion of missing information; see Supplementary Table S1) to infer phylogenetic relationships of Sumatran ranids that possess larvae with an abdominal sucker. Both maximum likelihood (ML) and Bayesian inference (BI) trees revealed the similar topologies for the relationships of ranid frogs with gastromyzophorous tadpoles (blue color in Fig. 1; original trees from both analyses provided in Supplementary Figs. S2–S3), with slight differences in the arrangement of terminal nodes (see Supplementary Figs. S2–S3). In the inferred phylogenies, *Sumaterana* was placed as sister taxon of *Clinotarsus* (BS/PP = 19/0.61; BS = 19) and *Wijayarana sumatrana* was closely related to *W.* sp3 from Java (BS/PP = 100/0.99; BS = 100). Minor differences between ML and BI trees also appeared in the intra-specific relationship of *Meristogenys amoropalamus* from Borneo. In the BI tree (Supplementary Fig. S2), *M. amoropalamus* was sister to a clade of *M. orphocnemis* + *M. poecilus*, whereas in the ML tree this species was sister taxon of an unidentified *Meristogenys* species (Supplementary Fig. S3). Although the mainland Asian genus *Amolops* also possesses gastromyzophorous tadpoles, both trees suggest *Amolops* to be closely related to ranid species that do not possess these specialized tadpole types (e.g., *Odorrana, Chalcorana, Pulchrana. Hylarana*), rather than grouping with *Huia*, *Meristogenys*, *Sumaterana,* and *Wijayarana* that have this larval form. In both analyses (Fig. S2), *Clinotarsus*, a ranid taxon with non-gastromyzophorous tadpoles was recovered as nested within the clade of Sundaland ranids with gastromyzophorous tadpoles (*Huia*, *Meristogenys*, *Sumaterana, Wijayarana*), however, with low support values.

### Sumatran paleodrainage systems and the distribution patterns of Sumatran ranids with gastromyzophorous tadpoles

**Figure 2:**
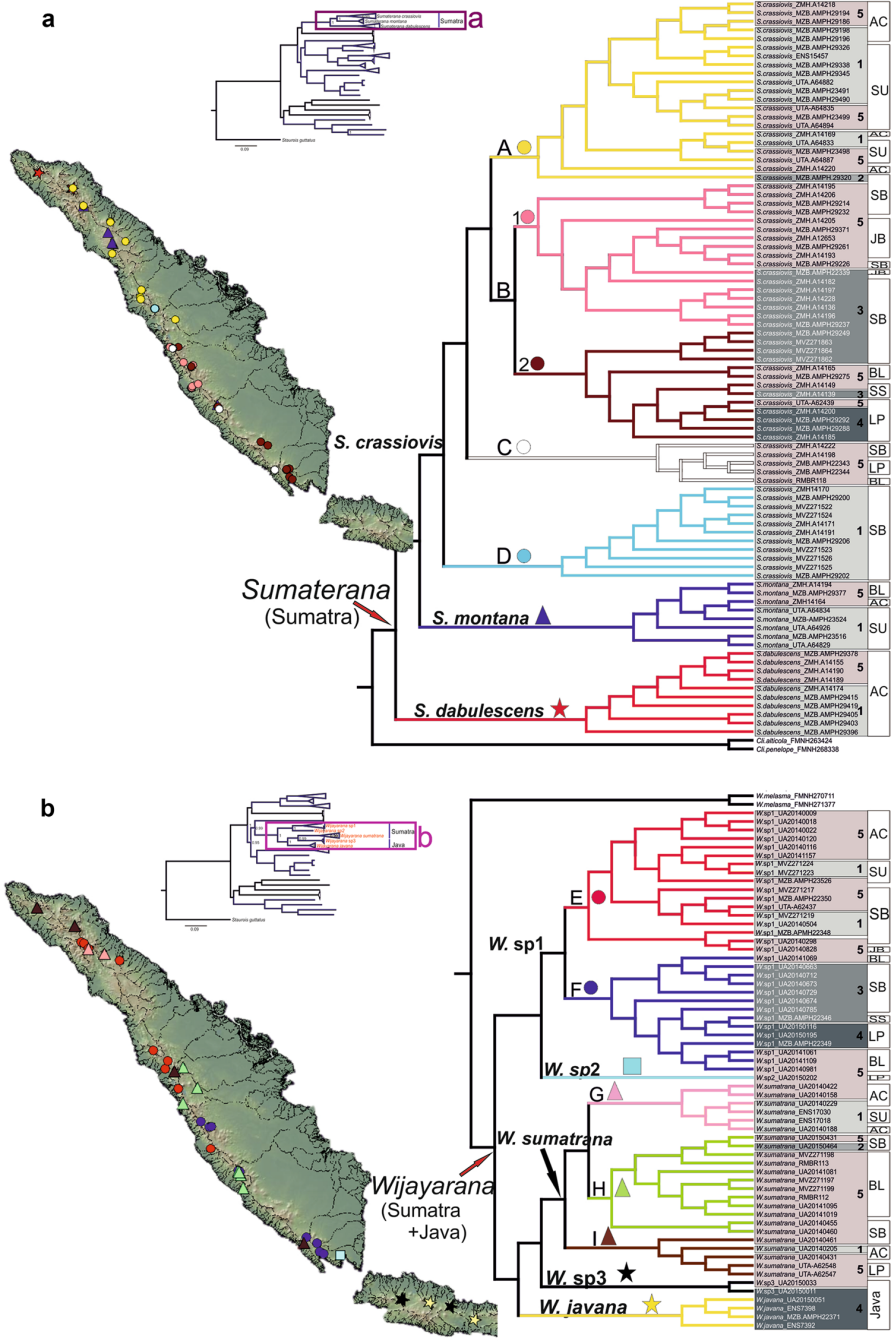
Distribution of *Sumaterana* (**a**) and *Wijayarana* (**b**) mapped over watersheds occurring on the island of Sumatra created using GeoMapApp (www.geomapapp.org). Colors on each branch represent the sampling locality of their respective taxa: *Sumaterana crassiovis* (clade A–D: yellow, pink, brown, white, light blue circles), *S. montana* (dark blue triangles), *S. dabulescens* (red star), *W.* sp1 (clade E–F, red and dark-blue circles), *W.* sp2 (light blue square), *W. sumatrana* (cade G–I: pink, green, brown triangles), *W.* sp3 (black star), *W. javana* (yellow star). Watersheds are color-coded and numbered with 1–5: 1 (Malacca Strait River System), 2 (Siam River System), 3 (North Sunda River System), 4 (East Sunda River System), 5 (watersheds that run into the Indian Ocean). Provinces on Sumatra indicated by AC (Aceh), SU (Sumatera Utara), SB (Sumatera Barat), JB (Jambi), BL (Bengkulu), SS (Sumatera Selatan), LP (Lampung).

We used the same BI tree (Fig. 1) to map the distribution of genetic variation of *Wijayarana* (*Wijayarana* sp1, *Wijayarana* sp2, *Wijayarana* sp3, *W. sumatrana*) and *Sumaterana* (*S. crassiovis*, *S. dabulescens*, *S. montana*) onto the respective watershed systems in Sumatra (Fig. 2a for *Sumaterana* and Fig. 2b for *Wijayarana*).

The phylogeographic structure of *Sumaterana* and *Wijayarana* did not show any clear correlation to the paleodrainage systems on Sumatra (Fig. 2a, b; see “Methods” for paleodrainage systems definition). Members of both genera from each paleodrainage system (sensu Voris^19^) east of the Barisan Mountain range were more closely related to members from their respective western watershed than to each other. Although Clade D shows a monophyletic group of samples from the Malacca Strait paleodrainage system, all samples were collected from different tributaries but within the same watershed (Sungai Rokan).

**Figure 3:**
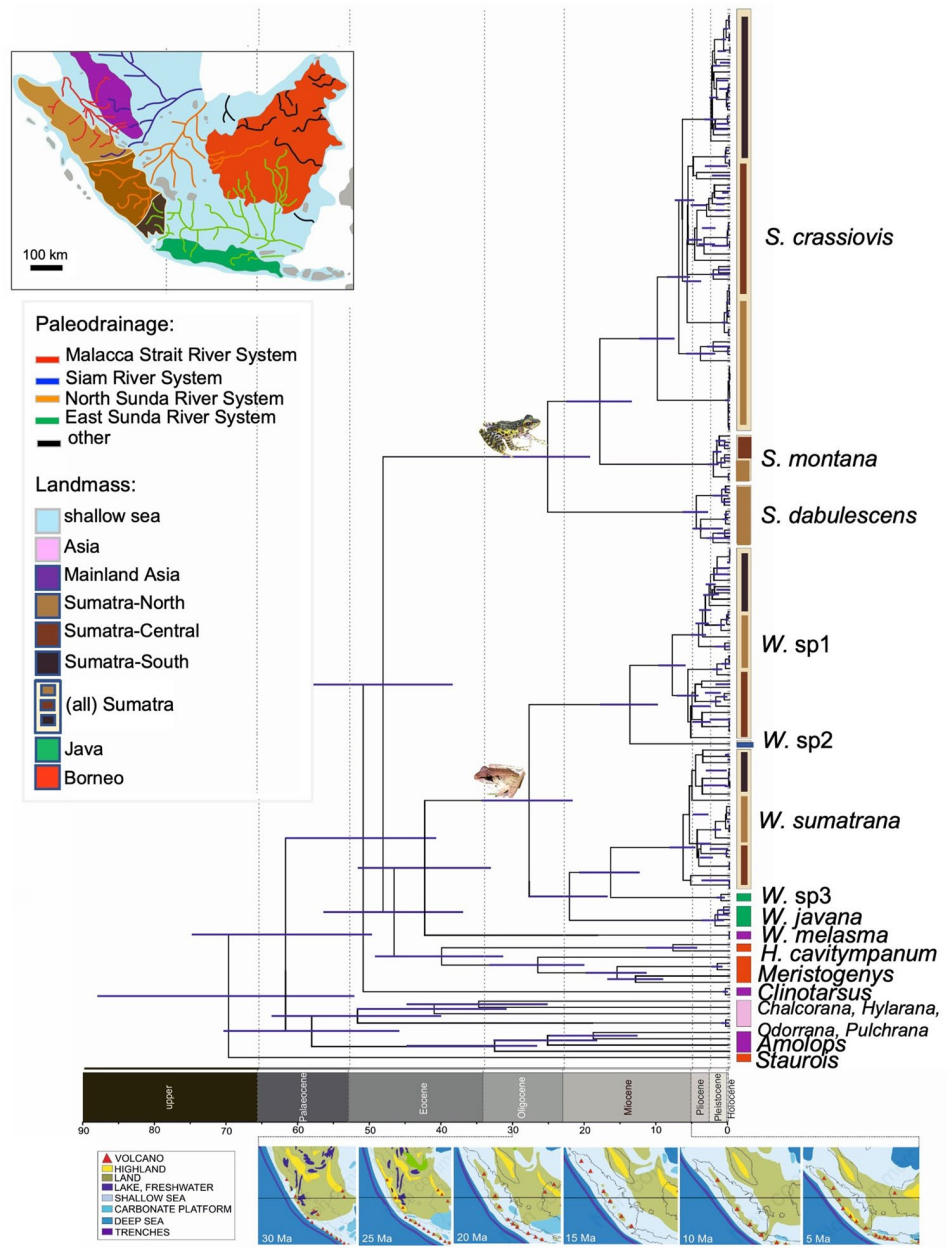
Divergence time estimates of *Sumaterana* and *Wijayarana* from Sumatra and Java (photos by UA). Colors and labels are explained in the legend in the bottom left box (sensu Hall^14^). Ancestral areas (circles at nodes) and geographic distributions (squares at tips) are color-coded according to the Pleistocene river systems in Sundaland (modified from Voris^19^).

Considering their wide distribution across the island, we used *Sumaterana crassiovis* (Fig. 2a Clade A–D)*, Wijayarana* sp1 (Fig. 2b Clade E–F), and *W. sumatrana* (Fig. 2b Clade G–I) to hypothesize divergence scenarios in Sumatra. The three taxa are genetically structured into northern (Sumatra-North in Fig. 3) and southern (Sumatra-Central and Sumatra-South in Fig. 3) lineages. Clade A, E, and G are distributed in the northern part, whereas clades B2, C, F, H, and I exhibit southern distributions. The Sumatera Barat province (located in the center of the island and comprising parts of Sumatra-North and Sumatra-Central in Fig. 3) appears to be the zone of range overlap between northern and southern species distributions. Samples from the Sumatera Barat (SB) province occur in both groups. This is supported by the fact that clade B1, which comprises samples from the SB province, also contains samples from Jambi (JB) province. Clade I encompasses samples from Aceh (AC), Sumatera Barat (SB), and Lampung (LP) provinces.

### Divergence time estimation and ancestral area reconstruction

We estimated divergence times of Sumatran frogs with gastromyzophorous tadpoles and their close ranid relatives to understand how the diversification history of these frogs relates to past geographic or climatic events on Sumatra. The chronogram (Fig. 3) resulting from this analysis differed slightly from the ML and BI trees in the arrangement of the terminal nodes (particularly for *Wijayarana* and *Sumaterana*). Furthermore, in the chronogram, *Clinotarsus* was placed as sister to a clade comprising taxa with gastromyzophorous tadpoles from Sundaland (*Huia* + *Meristogenys* + *Sumaterana* + *Wijayarana*) as opposed to being sister to *Sumaterana* in our ML and BI phylogenies.

**Figure 4:**
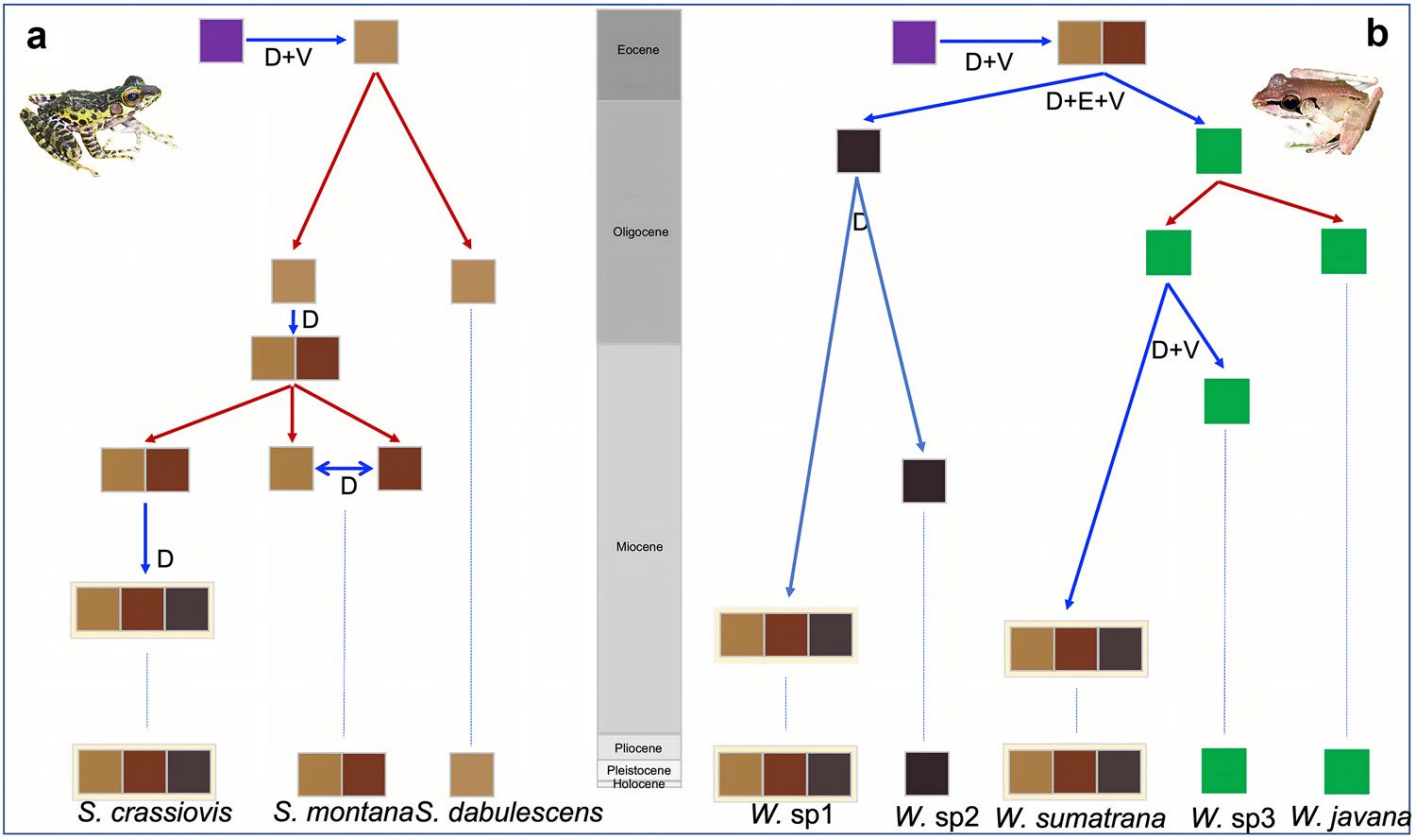
Depiction of the best-fit biogeographical model (BayAreaLIKE + J) as indicated by BioGeoBEARS for *Sumaterana* (**a**) and *Wijayarana* (**b**); photos by UA. Red arrows indicate range-copying while blue arrows represent Dispersal (D), Vicariance (V), and/or Extinction (E) events. Boxes with color represent geographic regions (see Fig. 3 for legend).

Our inference of biogeographic history of both genera suggest that the clade comprising *Huia* + *Meristogenys* + *Sumaterana* + *Wijayarana* began to diversify in the Early Eocene (approximately 47.75 Ma, Fig. 3) on Sundaland. While the MRCA of *Sumaterana* and *Wijayarana* emerged on Sumatra around the same time (Mid-Oligocene), the former continued to diversify in-situ on Sumatra with two major cladogenetic events at approximately 17.93 Ma and 10.01 Ma. The latter (the MRCA of *Wijayarana*) split into a Sumatran and a Javan lineage (~ 27.65 Ma; see Figs. 3 and 4). After initial in-situ diversification at approximately 22.11 Ma, this Javan lineage of *Wijayarana* went on to disperse back into Sumatra approximately 16.42 Ma, eventually becoming very divergent from its Javan members. On Sumatra, the original Sumatran lineage of *Wijayarana* diverged into *Wijayarana* sp1 and *Wijayarana* sp2 at approximately 13.78 Ma. Since the Early Pliocene (~ 5 Ma), both *Sumaterana* and *Wijayarana* have continued to diverge into many intra-specific lineages (Fig. 3).

## Discussion

The biodiversity and diversification patterns of amphibians on Sumatra and most of the Sundaland in general, remain poorly understood^5,39,41^, despite a variety of phylogenetic and taxonomic studies in the last decade^39,42–46^. Here, we add to this existing knowledge by analyzing the evolutionary relationships and biogeography of two stream-adapted genera of Sumatran frogs using multi-locus genetic data and comprehensive sampling. In doing so, we corroborate the recognition of *Wijayarana* as a distinct genus with at least five independent lineages^39,47^. This discovery is consistent with the evidence that Sumatran amphibian diversity is gravely underestimated (see Figs. 2 and 3) and that cataloguing of species diversity in Sumatra would benefit greatly if more thorough sampling efforts were performed^39,47–49^. Furthermore, the establishment of a stable taxonomy for Sumatran ranids with gastromyzophorous tadpoles (*Sumaterana* and *Wijayarana*), combined with the meticulous documentation of their geographic distribution in Sumatra represent important steps towards a better understanding of regional diversification patterns and processes^50,51^.

The presence of the Pleistocene paleodrainage system in Sundaland has been proposed as one of the main factors driving diversification patterns in some taxa (e.g., Sundaland freshwater fish^12^ and Mekong mud snakes^24^). However, our results failed to garner evidence for paleodrainages sensu Voris^19^ having significantly influenced evolution of Sumatran frogs *Sumaterana* and *Wijayarana* (Fig. 2), despite their strong dependence on rivers as larvae. In fact, the major diversification events in these frog lineages pre-date the formation of the paleodrainages by several million years (Fig. 3). Unlike previous studies, we also considered watershed systems that currently exist on the western slopes of the Barisan range in our biogeographic model (labeled with number 5 in Fig. 2). In this context, frogs from the eastern slopes of the Barisan Mountain range appear more closely related to individuals from the corresponding western slopes, despite belonging to distinct contemporary basins, which drain in opposite direction (i.e., west vs. east) (Fig. 2, Supplementary Table S1).

Unlike the freshwater fishes and the Mekong mud snakes that are restricted to fresh water, frogs with gastromyzophorous tadpoles presumably have two potential dispersal pathways: (1) via rivers during their larval stage and (2) via land during their adult stage. This implies that the dynamics of their spatio-temporal evolution are more complex than that of an organism with more restrictive means of dispersal (e.g., fish). Davis et al.^23^ proposed that both distance and potential barriers to gene flow may shape the genetic variation of taxa. Building on this premise and based on our results, we hypothesized that the formation and configuration of the Barisan Mountain range (e.g., contour and topology) probably have had the greater influence as drivers of diversification in this group of frogs.

Overall, the diversification of frogs examined herein seems largely structured geographically along a north–south axis (i.e., the orientation of the Bukit Barisan mountain range) within Sumatra (Fig. 2). Members of the genus *Sumaterana,* for example, display a spatio-temporal diversification pattern that appears to advance from the north of the island towards its southern end. A similar phylogeographic scheme also presents itself in the results of de Bruyn et al.^3^ who interpret this scenario as evidence for their “radiation by paleodrainage” diversification hypothesis for two Southeast Asian fish genera. However, the occurrence of this pattern of radiation in two vastly different local organisms (i.e., fish and frogs) at two vastly disparate temporal junctions (i.e., early Miocene vs. early Eocene onwards) indicates that the drivers of this phenomenon may be more numerous and more complex than just the configuration of Pleistocene River drainage systems.

The distribution boundary between the northern and southern groups appears to be located in the Sumatera Barat province (Fig. 2). However, some of the samples from this hypothesized boundary grouped either in northern or in southern lineages. Similar north–south genetic partitioning has also been reported for three species of Sumatran tree frogs of the genus *Rhacophorus* (*R. catamitus, R. modestus, R. poecilonotus*^13,52^), namely a northern clade encompassing Aceh and Sumatera Barat provinces (approximately the region north of Mount Kerinci) and a southern clade occupying West Sumatra and Lampung provinces (approximately all regions below Mount Kerinci). Additionally, Whitten^53^ demonstrated that the Toba region is the zoogeographic boundary between several monkey species: *Presbytis thomasi* and *Hylobates lar* in the north, and *Cephalopachus bancanus* and *Tapirus indicus* in the south. These results are also corroborated by at least one more phylogeographic study on Sumatran orangutans^54^. Whitten^53^ suggested that Toba super volcanic eruption (~ 74 k year)^55,56^ may have influenced the population structure of these monkey species^54^ and facilitated re-colonization on Sumatra^57^. However, O’Connell et al.^52^ argued that the partition between the northern and southern populations of the *Rhacophorus* species was driven by the paleoclimate during the Miocene and Pleistocene epoch.

A similar diversification process, with a slight variation, is also presented by Shaney et al.^58^ for Sumatran montane dragons (genera *Dendragama, Lophocalotes,* and *Pseudocalotes*) wherein the lizards are genetically segregated into four major biographic zones: North, North-Central, Central, and South montane zones. Elevational boundaries, the paleoclimate during the Pleistocene, and ecological factors (e.g., competitive niche exclusion) have all been suggested as likely drivers of phylogenetic structuring in these agamid genera^58^. Thus, it remains hard to state with confidence whether the population genetic structuring shown by *Sumaterana* and *Wijayarana* (north–south partitioning) was solely influenced by the paleoclimate and geology of Sumatra (as in *Rhacophorus*^52^), or also influenced by other factors (e.g., elevation, ecology as proposed by Shaney et al.^58^). Given existing data, it is also unclear if the Toba eruption played a decisive role in the aforementioned spatio-temporal evolutionary dynamics of our focal taxa. Sumatra has had an extremely active and complex tectonic history and several local volcanic eruptions may have played a role the genetic partition and recolonization of regional species.

In the hypothetical scenarios of in-situ diversification of the Sumatran *Rhacophorus,* O’Connell et al.^52^ suggested that the northern populations of these tree frogs were considered to have occupied the northern region of Sumatra at the beginning in the Miocene^52^ when suitable habitats were likely to be abundant in the region^7^. According to our dated phylogeny (Fig. 3) and ancestral area reconstruction (Fig. 4), this ‘northern route’ hypothesis may also apply for *Sumaterana* and *Wijayarana*, which initially colonized Sumatra at approximately 25.11 Ma and 27.65 Ma, respectively. Notably, our analyses suggested that *Wijayarana* recolonized Sumatra again at approximately 16.42 Ma (this time from Java to Sumatra; see Figs. 3 and 4). These time estimations marked the beginning of in-situ diversification of the two genera on the island of Sumatra, which presumably occurred synchronously.

Hall^14,16^ suggested that by the Mid-Late Oligocene major parts of Sundaland were subaerial including some freshwater lakes. During this time, the northern part of Sumatra had a more seasonal climate^8^ and lacked active volcanoes^14,16^. In contrast, the southern part of the island had a wet climate and harbored several active volcanoes. Consequently, habitat types in the northern and southern parts of Sumatra were very different. Although volcanoes were not present at the time in the northern region, some highlands, which provide ample cascading habitats in this area, were known to be present^16^. Our dated phylogeny and ancestral area reconstruction analyses suggest that the population of the most recent common ancestor (MRCA) of *Sumaterana* first evolved in cascading habitats of northern Sumatra (light brown color in the map; Fig. 3) after initial colonization from mainland at approximately 25.11 Ma. Considering some overlapping distributions of the three extant *Sumaterana* species, the MRCA of *S. dabulescens* and the MRCA of *S. montana* and *S. crassiovis* adapted to distinct but overlapping elevational ranges, thus occurring in sympatry in some areas (see Arifin et al.^39^). In contrast, at ~ 27.65 Ma the MRCA of *Wijayarana* were migrated to Java from mainland Asia via Sumatra, subsequently experiencing an extinction event in its north and central ranges of Sumatra (see Fig. 4).

Between 20–15 Ma, the chain of volcanoes in Sumatra shifted northwest up to the northern part of Sumatra due to strike-slip faulting, and in response to the region-wide Sundaland deformation initiated by Australian plate’s collision with eastern Indonesia^14,16^. The increase of regional marine transgression resulted in increased sea levels and therefore large parts of Sumatra were gradually submerged^14,59^, leaving only high elevation mountains above water. Our ancestral area reconstruction suggests that the MRCA of *Sumaterana dabulescens* and the MRCA of *S. montana* and *S. crassiovis* (Fig. 3) could have been locally concentrated on different volcanic peaks serving as refugia on northern Sumatra and thus becoming geographically isolated from each other. The ever-wet climate across the whole island during this time^8^ may have supported populations in limited numbers on mountain tops, impeding gene flow between isolated refugial populations. The same conditions may have applied to the MRCA of *Wijayarana*, which presumably occupied available refugia on mountain tops across the Miocene highlands of south Sumatra. On the other hand, the tectonic activity that shifted the position of the western-most end of Java to connect with the southernmost tip of Sumatra^14,16^, putatively allowed the MRCA of the Javan *Wijayarana* to colonize Sumatra at approximately 16.42 Ma onwards. This lineage eventually gave rise to *W. sumatrana* via vicariance due to the isolation enforced by a marine barrier. Around the same time, ancestral populations of Sumatran *Wijayarana* (Fig. 2) from the mountain-top refugia of southern Sumatra dispersed into the more central regions of the island.

As sea levels gradually decreased^16^ from 10 Ma onwards, gene flow between previously geographically isolated populations could potentially have resumed. The MRCA of *Sumaterana crassiovis* could have then migrated further south along with the MRCA of *S. montana*, whereas the MRCA of *S. dabulescens* remained in the north. In contrast, the MRCA of *Wijayarana sumatrana* and the MRCA of *W.* sp1 and *W.* sp2 moved northwards occupying all suitable niches on the island. From 5 Ma (the beginning of the Pliocene) onwards, Sumatra experienced further land accretion, which dramatically modified the topology of the island. The number of volcanoes increased, leading to a greater number of high-altitude habitats in southern Sumatra^14,16^. This geological process became more frequent during the Pleistocene and continued into the Holocene in many areas^60^. Seasonal climate occurred during this period^8^. Consequently, suitable cascading habitats for both *Sumaterana* and *Wijayarana* became more abundant across the island. Our overall results support the premise that the gradient of divergent environments between the northern and southern parts of Sumatra during the Pliocene onwards initiated the segregation of Sumatran fauna (e.g., *Sumaterana* and *Wijayarana*) into the northern and southern groups seen today (see Figs. 2 and 3). These environmental conditions could have been effective drivers of synchronous (in-situ) diversification within the two genera. The diversification became more pronounced from the beginning of the Pliocene onwards, compared to the previous epoch (see Fig. 3).

In conclusion, our work is the first to leverage contemporary biogeographic approaches to elucidate the evolution of these Sumatran ranids with gastromyzophorous tadpoles. In doing so, our study sheds light on the diversification pattern of *Sumaterana* and *Wijayarana* on the island of Sumatra through space and time. The results of our study show that both *Sumaterana* and *Wijayarana* probably experienced rapid divergence from the Late Miocene or the Early Pliocene onwards (Fig. 3) driven primarily by the paleo-climatic (cooling and warming) and changing of land and sea level during this period^8,14,16^. However, we would also like to highlight that there are several caveats concerning this interpretation given the complex climatic and geological history as well as the lack of an adequate number of comparative studies for the region. Considering current distribution patterns for both genera, elevational gradients might have also been influential^58^^; *unpubl*.^. Furthermore, ecological factors (e.g., competitive exclusion) may have also been drivers for the observed genetic segregation. For example, during our fieldwork, we often found *Sumaterana* and *Wijayarana* living in sympatry near the same stream. Nevertheless, *Wijayarana* tend to avoid the water whenever *Sumaterana* species were found in the stream^*pers. obs*^. Observation such as these indicate that the current evolutionary information on these ranids, would benefit greatly by having more complementary ecological data. Evolutionary and ecological investigations will both be vital for a clearer understanding of the complex biogeography of not only these unique amphibians but also of other Sundaland fauna in general.

## Methods

### Taxon sampling and molecular data

We sampled a total of 146 ranid frogs belonging to the genera *Sumaterana* (N = 85) and *Wijayarana* (N = 61) along cascading habitats in the island of Sumatra and Java in 2008 and between 2013 and 2016. Samples were obtained from 55 sampling points for *Sumaterana* and 48 sampling points for *Wijayarana* (N = 1–4 samples per site) along the Sumatran and Javan transect (Supplementary Fig. S1, Supplementary Table S1). Sampling sites comprise four Sundaland paleodrainage systems east of the Barisan Mountain range (sensu Voris^19^) and also current watershed systems that run westward towards the Indian Ocean. All methods performed in this study (e.g., collecting, handling, and euthanizing specimens) were performed in accordance with the relevant guidelines and regulations approved by the UTA Institutional Animal Care and Use Committee (IACUC; number UTA IACUC A12.004) and by the Federal Office of Justice in Germany (Tierschutzgesetz, https://www.gesetze-im-internet.de/tierschg/BJNR012770972.html). Frogs were collected by hand and the muscle or liver tissues were preserved either in ethanol (96%), RNAlater (Sigma Aldrich, USA) or lysis buffer (0.5 M Tris / 0.25% EDTA / 2.5% SDS, pH 8.2) for DNA analyses. Specimens were fixed in 4% neutral-buffered formalin and then transferred to 70% ethanol for long-term storage. Additionally, we included samples (N = 13) from other ranids with gastromyzophorous larvae from Borneo, and mainland Asia as outgroups: *Amolops afghanus*, N = 1; *A. marmoratus*, N = 1; *A. indoburmanensis*, N = 1; *A. panhai*, N = 1; *H. cavitympanum*, N = 2; *Wijayarana melasma*, N = 2; *Meristogenys amoropalamus*, N = 1; *M. kinabaluensis*, N = 1; *M. orphocnemis*, N = 1; *M. poecilus*, N = 1; *M.* sp., N = 1; and other closely related ranids (N = 7): *Chalcorana chalconota*, N = 1; *Clinotarsus penelope*, N = 1; *Cli. alticola*, N = 1; *Hylarana erythraea*, N = 1; *Odorrana hosii*, N = 2; *Pulchrana picturata*, N = 1). *Staurois guttatus* (N = 1) was selected to root the trees^61^. All specimens are deposited in one of the following museums: the California Academy of Sciences (CAS), San Francisco, USA; Museum Zoologicum Bogoriense (MZB), Bogor, Indonesia; Zoologisches Museum Hamburg (ZMH), Hamburg, Germany; Museum of the University of Texas Arlington (UTA), Arlington, USA; Museum of Vertebrate Zoology (MVZ), Berkeley, USA; and Field Museum of Natural History (FMNH), Chicago, Illinois USA. Detailed information of the specimens used in this study (taxon name, voucher number, locality, GPS coordinates, elevation, and watershed information) is available in Supplementary Table S1.

DNA extraction, PCR, and sequencing followed published protocols^39^. Sequences of ten loci were generated for five mtDNA (12S, 16S + tRNA^val^, COI, cyt b, ND2) and five nucDNA (Brain-derived neurotrophic factor, BDNF; Neurotrophin 3, NTF3; Proopiomelanocortin, POMC; recombination-activating gene 1, RAG1; tyrosinase exon 1, TYR) markers; see Supplementary Table S1 for Genbank accession numbers. Primer information and PCR annealing temperatures applied for this study are provided in Supplementary Table S2.

### Phylogenetic analyses

The dataset of ten concatenated genes consisted of 7,582 bp. In order to create a dataset with a minimum amount of missing data, we only used individuals for which at least three loci (or 1,942 bp total length) were successfully sequenced (see Supplementary Table S1 for details on marker coverage for each sample). Our final dataset comprised 146 specimens for *Sumaterana* (N = 85) and *Wijayarana* (N = 61), and an additional 21 sequences for closely related taxa. We tested a variety of models and partitioning strategies to find the best partitioning scheme and substitution models for the concatenated dataset using PartitionFinder v.1.1^62^ and the Bayesian Information Criterion (BIC), which suggested using eleven partitions (Supplementary Table S3).

We used two different reconstruction methods to generate phylogenetic trees: maximum likelihood (ML) with RAxML v. 8.2.10 (Stamatakis^63^) and Bayesian inference (BI) with MrBayes v.3.2.6^64,65^ using the CIPRES Science Gateway v.3.3^66^. In RAxML, eleven distinct partitions and associated models (GTR + Γ + G) were defined, and we performed joint branch length optimization. Tree support was obtained running 1,000 bootstrap replicates.

For the Bayesian analysis, we performed two independent runs with one cold and three heated chains for 50 million Markov Chain Monte Carlo (MCMC) iterations, sampling every 1,000 generations. Convergence of runs was assessed using the trace plot generated from MrBayes, the average split frequencies being < 0.01, and by assessing ESS values (> 200) of the log files with Tracer v.1.6^67^ after discarding the first 25% of samples as burn-in. FigTree v.1.4.3 (http://tree.bio.ed.ac.uk/software/figtree/) was used to visualize the 50% majority consensus trees from RAxML and MrBayes. Strong support^68,69^ was defined by nodal support with bootstrap values (BS) ≥ 70 for the ML tree^69^ and posterior probability (PP) ≥ 0.95 for the Bayesian analyses^70^.

### Mapping of distribution patterns

The BI tree was transformed and edited in MESQUITE v.3.6^71^ into a color-coded tree to map the distribution patterns of the Sumatran ranid frogs with gastromyzophorous tadpoles. We followed Voris’^19^ definition of the four paleodrainage systems in Sundaland (color-coded and labeled with number 1–4 in Fig. 2). Voris^19^ did not specify any watersheds that ran into the Indian Ocean (western slope of the Bukit Barisan mountain range) in his paleodrainage definition. Our samples were also collected from both the western and eastern slopes of the Bukit Barisan mountain range. Thus, in this study, we categorized all watersheds on the western slopes of the mountains as distinct drainage system (color-coded and labeled with number 5 in Fig. 2).

### Divergence time estimation

We estimated divergence times of Sumatran ranids with gastromyzophorous tadpoles using BEAST v.2.4.8^72^. We performed analyses with the complete dataset (N = 167) and the partitioning scheme as described in Supplementary Table S3. The molecular clock was calibrated using two evolutionary rates from previous studies: (1) 1.41% (0.705% per lineage)/MY, a rate estimated for the cytb region of *Sylvirana latouchii*^73^, another ranid species; and (2) 1.00% (0.50% per lineage)/MY, as suggested by Kakehashi et al.^74^ for the 16S region of anurans more generally. Though we used the same values for the mean and standard deviation as in Tominaga et al.^75^ the prior distribution was set to a uniform distribution rather than a normal one (compared to Tominaga et al.^75^) because the analyses failed to reach convergence under a normal distribution. First, we performed a series of test runs to find the optimal settings for our data. We varied the clock model, tree prior, and gamma hyperparameter and performed runs while retaining the remaining parameters as default. Each preliminary run was performed for 100 million iterations, sampling every 10,000 generations. We then compared the log files and chose the parameter set with the highest likelihood for our final analysis (see Supplementary Table S4 for likelihood comparison). The best parameter set was as follows: uncorrelated log-normal relaxed clock model, Yule tree prior, and HKY for the substitution model with four gamma categories. We performed the final analysis in two independent runs of Markov chains for 500 million generations, sampling every 10,000 generations. We used Tracer v.1.6^67^ to evaluate stationarity of the Markov chain and potential autocorrelation (effective sample sizes > 200). The first 25% of samples were discarded as burn-in, and the samples of both runs were combined with LogCombiner v.2.4.8^72^. TreeAnnotator v.2.4.8^72^ was used to identify and annotate the maximum clade credibility tree.

### Ancestral area reconstruction

We performed statistical ancestral area reconstruction for the taxa of interest using BioGeoBEARS^76^ as implemented in RASP 4.2^77^. We pruned our calibrated trees from BEAST to obtain a mOTU-based tree for this analysis. We labeled each sample with their respective geographic distributions: A (Sumatra-North, comprises all samples from the Malacca Strait and Siam river systems including one from their neighboring watershed in the western slopes of Bukit Barisan), B (Sumatra-Central, comprises all samples from the North Sunda river system including one from their neighboring watershed in the western slopes of Bukit Barisan), C (Sumatra-South, comprises all samples from the East Sunda river system including one from their neighboring watershed in the western slopes of Bukit Barisan), D (Java), E (Borneo), F (Asia). We ran the analysis using the BayAreaLIKE + J model, which was identified as optimal during model selection by the program, set the number of maximum areas at each node to six, while leaving all other settings at default values.

## Supplementary Information

Supplementary Information 1.

Supplementary Information 2.

## Data Availability

Data generated for this study is available in Supplementary information. Sequences are deposited in GenBank with accession numbers provided in Table S1.
