# Supplementary material for: Phylogeographic inference of Sumatran ranids bearing gastromyzophorous tadpoles with regard to the Pleistocene drainage systems of Sundaland
Source: Sci Rep. 2022 Jul 19;12:12013. doi: 10.1038/s41598-022-14722-9 (PMC9296532; doi:10.1038/s41598-022-14722-9)
Supplement: Supplementary file 1 — Supplementary Information 1. [file 41598_2022_14722_MOESM1_ESM.pdf]

Supplementary Figures

Phylogeographic inference of Sumatran ranids bearing gastromyzophorous tadpoles with regard to the Pleistocene drainage systems of Sundaland

Umilaela Arifin, Utpal Smart, Martin Husemann, Stefan Hertwig, Eric N. Smith, Djoko T. Iskandar, and Alexander Haas

Author for correspondence: Umilaela Arifin, Leibniz Institute for the Analysis of Biodiversity Change, Centre for Taxonomy and Morphology, Martin-Luther-King-Platz 3, 20146 Hamburg, Germany. Email: u.arifin@leibniz-lib.de

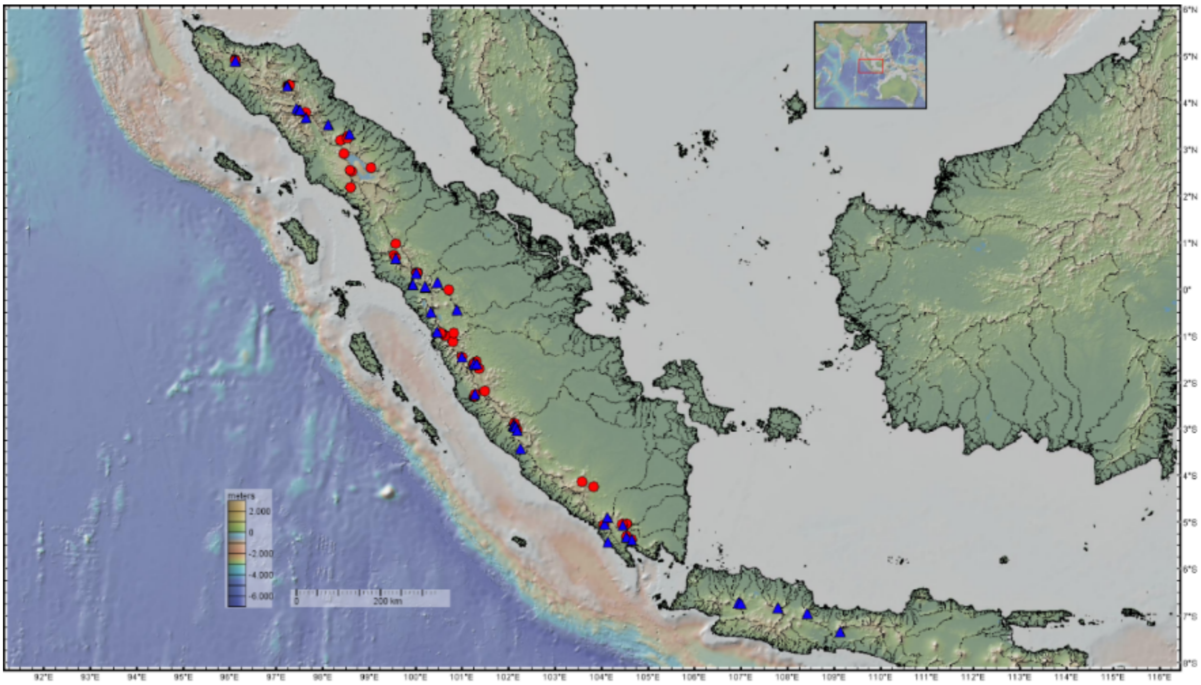

Figure S1 Map of sampling localities of the genera *Sumaterana* and *Wijayarana* used in this study. Map generated using GeoMapApp (www.geomapapp.org).

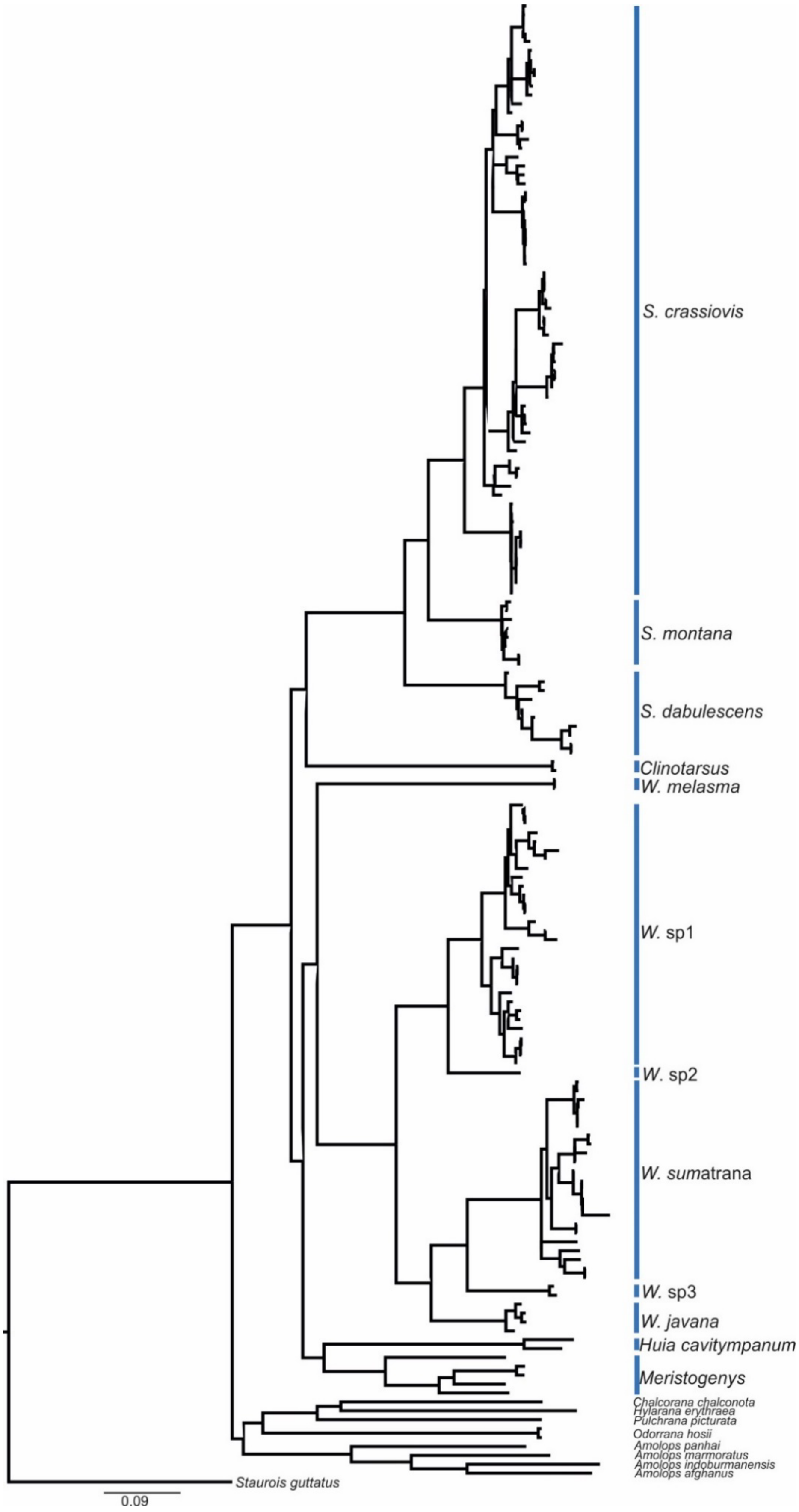

Figure S2 Original tree reconstruction using BI analysis

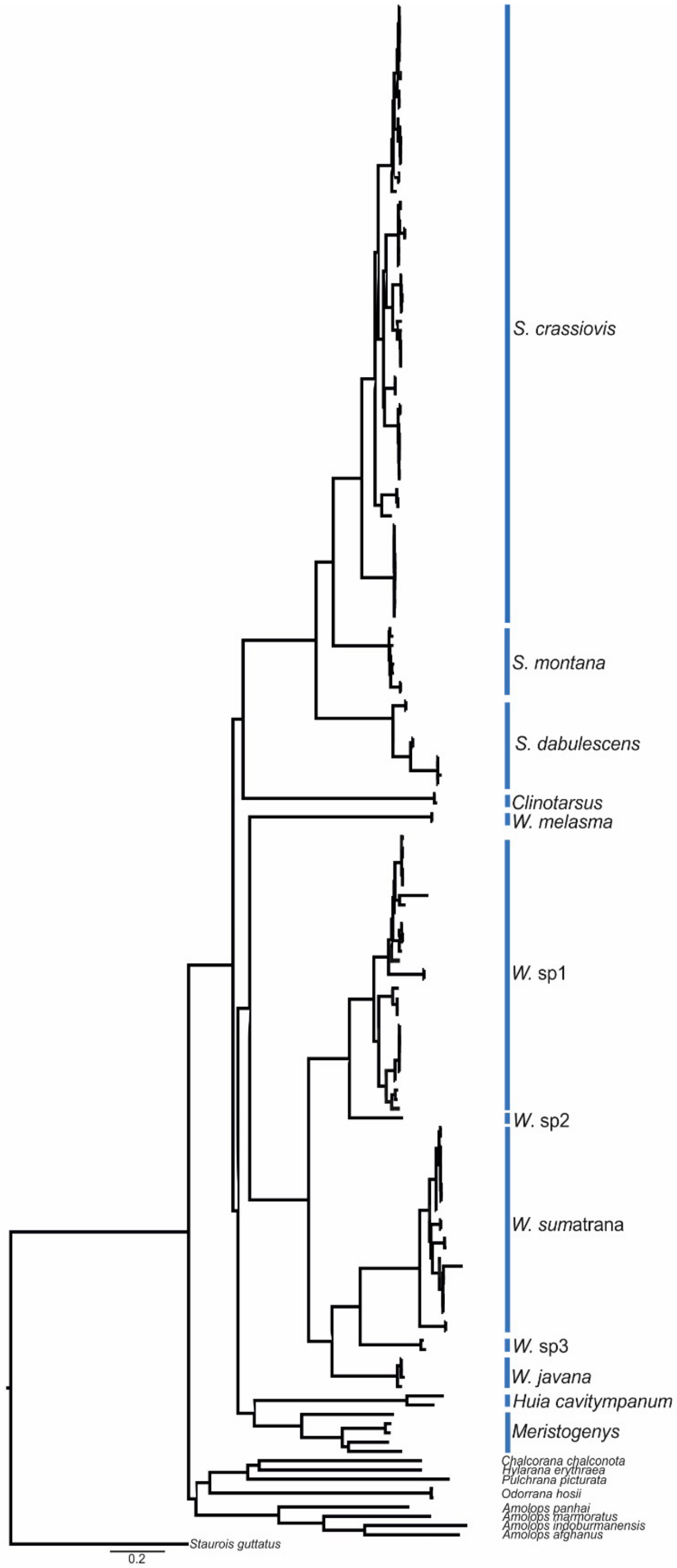

Figure S3 Original tree reconstruction using ML analysis.
